# Evaluation of USP 3 Apparatus to Develop Biopredictive Fasted and Fed Dissolution Methods for Extended-Release Desvenlafaxine Succinate Tablets

**DOI:** 10.1021/acsomega.5c11179

**Published:** 2026-02-19

**Authors:** Gustavo V. Carapeto, Beatriz C. Nunes, Marcelo D. Duque, Michele G. Issa, Humberto G. Ferraz

**Affiliations:** † Department of Pharmacy, School of Pharmaceutical Sciences, 28133 Universidade de São Paulo-USP, Av. Prof. Lineu Prestes 580, São Paulo 05508-080, SP, Brazil; ‡ Department of Pharmaceutical Sciences, Institute of Environmental, Chemical and Pharmaceutical Sciences, 505146 Universidade Federal de São Paulo-UNIFESP, Rua São Nicolau, 210 Centro, Diadema 09913-030, SP, Brazil

## Abstract

The development of distinct biopredictive methods for fasted and fed states using a physiologically based biopharmaceutics modeling (PBBM) approach is essential for accurately evaluating drug release from solid oral dosage forms, especially extended-release products. However, a fed state biopredictive method for desvenlafaxine tablets is not currently available. Hence, the study aimed to investigate the application of the USP 3 apparatus to develop biopredictive methods for desvenlafaxine tablets. Initially, an existing fasted state biopredictive USP 2 dissolution method was adapted to increase dissolution hydrodynamics by scaling the rotation speed to 75 and 100 rotations per minute (rpm). Subsequently, two dissolution methods were developed using the USP 3 apparatus to emulate fasted and fed conditions. The fed biopredictability of the methods was assessed using a previously developed GastroPlus model to simulate fed conditions under 800 kcal and 50% fat meal. Statistical analysis of dissolution profiles obtained in the paddle apparatus revealed no significant difference from the original 50 rpm method and lacked biopredictive for the fed state, indicating the unfeasibility of developing such a method in this apparatus. In contrast, USP 3 proved to be an important tool to develop a fed state method, since it was biopredictable based on simulation analysis. Additionally, no significant differences were observed between USP 3 methods employing pH-gradient media and those using 0.9% NaCl as the sole medium. These findings highlight a hydrodynamic-driven approach for applying USP 3 to develop distinct biopredictive dissolution methods for fasted and fed states, particularly for high-solubility drugs formulated in robust hypromellose matrices.

## Graphical abstract

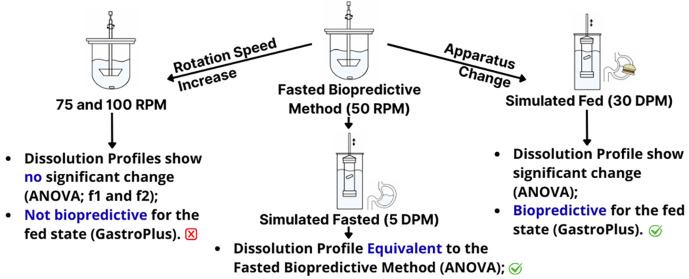

## 1 Introduction

An accurate evaluation of the drug product release characteristics is critical in the context of the development of extended-release dosage forms, given the high impact that physiological conditions can have on the dissolution process. A very well-known example is the difference between observed in vivo release and absorption of the same drug product in fasted and fed states.^1^

In this sense, the use of distinctive biopredictive dissolution methods for fasted and fed states leads to a superior formulation characterization, resulting in a much more robust analysis of the in vitro release profile of extended-release dosage forms, impacting both quality control and development of new drug products, based on the quality by design approach.^2,3^ Moreover, the use of modeling and simulation approaches, based on physiologically based biopharmaceutics modeling (PBBM), is a strong ally to reduce time and cost resources in the development of such methods.^4^

Biopredictive dissolution methods are described by Heimbach et al.^5^ as a method capable of obtaining dissolution profiles that can be used to predict pharmacokinetic (PK) profiles. In this sense, for a method to be considered biopredictive, its predicted systemic exposure must be comparable to available in vivo PK parameters.^6^ In this context, methods that have their biopredictability confirmed may be used in modeling and simulation software, such as GastroPlus, to predict bioequivalence outcomes of pharmaceutical products in development.^7^ However, it is important to note that a biopredictive method does not have to be biorelevant. A recent example is a case study discussed by Heimbach et al.^8^ in which a quality control method developed was biopredictive but not biorelevant.

Regarding the application of such methods, it is known that they may vary based on the class of the drug, according to the Biopharmaceutics Classification System (BCS) and the type of formulation. In this sense, Wu et al.^7^ considered that for BCS I and III immediate release formulations, PBBM may be used to widen dissolution specifications that may be too strict. For class II drugs, absorption is dissolution-limited, and therefore, prediction of clinically relevant data is key to the success of such products. Finally, for BCS IV, PBBM may identify if the absorption of a drug is more permeability-controlled than solubility-controlled and, therefore, modulate the defined dissolution specifications accordingly. In addition, controlled-release products especially benefited from biopredictive dissolution methods, as the release behavior directly impacts the PK parameters obtained in vivo.^9^

Within the dissolution apparatus available, USP 3, also known as the reciprocating cylinder, shows great advantages for this context, since it has great flexibility in terms of pH gradients and promotion of variations on media volumes, agitation speed, and retention time, leading to methods specifically designed to simulate gastrointestinal tract compartments, under fasted and fed conditions.^10,11^

Apart from the USP 3 apparatus, the USP 4, known as the flow-through cell apparatus, also stands out, as media pH changes can be easily performed, emulating gastrointestinal physiology,^12^ being especially beneficial for low solubility drugs.^13^ In the case of highly soluble drugs, the USP 4 closed system is the most suitable since it allows the test to be performed using small volumes of dissolution medium. On the other hand, compared to the USP 4 open system, USP 3 becomes more advantageous.^11^

Desvenlafaxine succinate is a high-solubility drug, meaning it is soluble throughout the whole physiological pH range. In addition, according to Franek et al.,^14^ when in extended-release tablets, it is considered a BCS class I drug. In this context, the USP 3 apparatus may be more beneficial in terms of dissolution media waste reduction.

Moreover, few studies have explored the use of PBBM-based software to evaluate and optimize dissolution methods developed on the USP 3 apparatus, aiming to obtain biopredictive conditions for both fasted and fed states. Within these studies, the authors focused on analyzing the impact of pH-gradient variations or comparing the use of traditional USP 3 with an adapted new proposed apparatus.^15,16^

Additionally, with regard to extended-release desvenlafaxine succinate tablets, the available information on dissolution methods is minimal. In a previous study conducted by our research group, a biopredictive dissolution method for the fasted state using the USP 2 apparatus was developed.^17^ However, a dissolution method that can be used to predict the fed state is still lacking in the literature.

Therefore, the main goal of this work was to obtain distinct biopredictive dissolution methods able to forecast the dissolution behavior of desvenlafaxine extended-release tablets under fasted and fed conditions while using a PBBM-based approach and evaluating the application of USP 2 and USP 3 apparatuses in this context. In addition, identifying the benefits of the USP 3 apparatus in obtaining such methods for high-solubility drugs is also part of the study.

## 2 Materials and Methods

### 2.1 Materials

Desvenlafaxine succinate monohydrate, 94.68% purity grade, was kindly provided by Aché Laboratórios Farmacêuticos S.A. and used as a work standard for drug quantification in the samples analyzed.

The chemical reagents employed for the preparation of the dissolution media, described further in Sections 2.5 and 2.4, were as follows: hydrochloric acid P.A. 37% (Mallinckrodt Chemical Co.) and sodium chloride P.A. 99% (Dinâmica, Brazil) for the pH 1.3 medium; sodium chloride P.A. 99% (Dinâmica, Brazil) for the NaCl 0.9% medium; and sodium hydroxide pellets P.A. 98% (Labsynth, Brazil), phosphoric acid P.A. 85% (Labsynth, Brazil), and anhydrous monobasic potassium phosphate P.A 99.32% (Neon, Brazil) for all other media.

Extended-release tablets containing 50 mg of desvenlafaxine, Pristiq (produced by Pfizer Ireland Pharmaceuticals and marketed and distributed in Brazil by Wyeth Indstria Farmacêutica Ltd.), were obtained and used in this study within their expiration date.

### 2.2 Workflow Diagram

The work performed in this study is summarized in the diagram presented in Figure 1.

**Figure 1:**
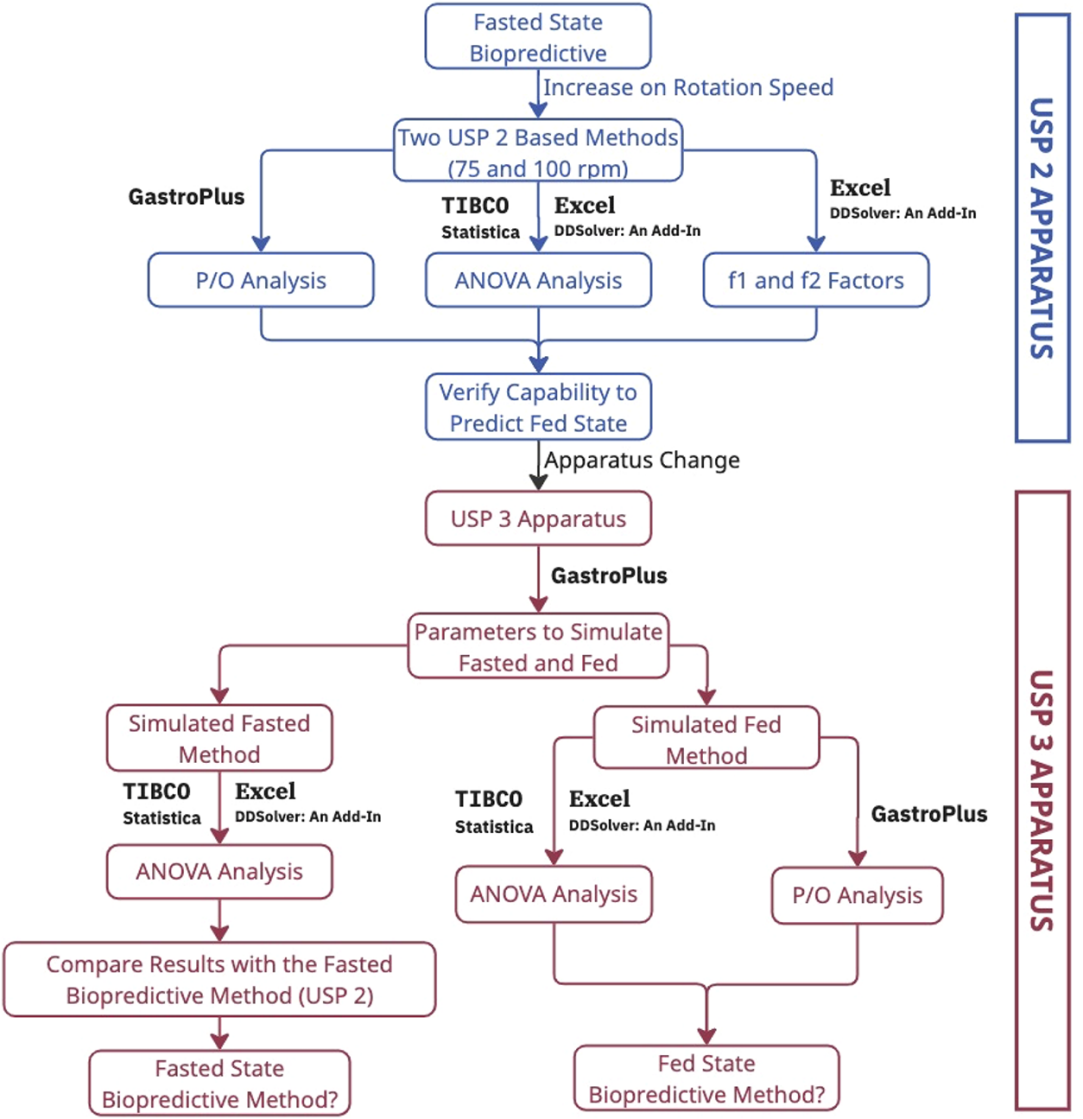
Graphical representation of the workflow of the present study.

### 2.3 Fed State Physiologically Based Biopharmaceutics Model

The model used to evaluate the biopredictive power of the dissolution methods using the USP 3 apparatus was the same as previously described by Carapeto et al.^17^ for the fasted state, using GastroPlus version 9.8.3. For the fed state, the effect of the administration with a meal was included in the model by selecting the Human Fed State, User Defined Calories and Fat (800 kcal and 50% fat) meal in the software.

The obtained dissolution profiles corresponding to each dissolution method, as described in the following section, were used as input data in GastroPlus. Weibull function was applied to each dissolution profile, and a population simulation was run considering *n* = 43 subjects with a mean body weight of 70 kg (CV% = 10). The predicted geometric mean values of the maximum plasma concentration (*C*_max_) and area under the curve (AUC0-*t*) were compared to the values obtained by Pedrazzoli-Júnior et al.^18^ for the fed state using the same number of subjects. The values of the predicted/observed ratio were considered adequate if they were between the range of 0.80 and 1.25.^19–21^

The biopredictive dissolution method (USP 2) for the fasted state previously described by our research group^17^ was evaluated considering the fed state to check its ability to be biopredictive for both conditions. Additionally, modifications of the same method, with higher rotation speed values (75 and 100 rpm), and USP 3 defined methods were also tested.

### 2.4 In Vitro USP 2 Dissolution Test

Dissolution tests, described in Table 1, were performed in triplicate based on the biopredictive method developed by our research group in a previous study,^17^ varying the rotation speed to 75 and 100 rpm. Dissolution media (900 mL) was previously degassed and heated to 37 °C by Ezfill+ (Distek Inc., North Brunswick, NJ), and 5 mL samples were collected (without fresh medium replacement) by a VK 8000 Automatic Dissolution Sampling Station (Agilent Technologies Inc., Santa Clara, CA), filtered through 45 μm polyethylene cannula filters (Rockwheel, Rio de Janeiro, Brazil) and analyzed by UV spectrophotometry at 223.5 nm.

**Table 1:** Description of Dissolution Methods Based on the USP 2 Apparatus Employed to Evaluate the Pristiq Tablets

method	apparatus	rotation speed	dissolution medium
USP 2/50 rpm/NaCl 0.9%^a^	USP 2 (paddle)	50 rpm	NaCl 0.9%
USP 2/75 rpm/NaCl 0.9%	USP 2 (paddle)	75 rpm	NaCl 0.9%
USP 2/100 rpm/NaCl 0.9%	USP 2 (paddle)	100 rpm	NaCl 0.9%

a Dissolution condition performed in sextuplicate.

### 2.5 In Vitro USP 3 Dissolution

Two physiological scenarios were tested: fasted and fed, with pH values and retention times in each vessel row (Table 2) defined by adapting the physiological gastrointestinal tract compartment information available on the ACAT model of GastroPlus software, version 9.8.3. Methods with the same retention times defined, but employing exclusively 0.9% NaCl as dissolution medium, were also tested.

**Table 2:** Compartments Simulated, Employed pH Values, and Retention Times on Each Vessel Row Used to Simulate Fasted and Fed State Conditions

	fasted state		fed state	
vessel row	retention time	compartments simulated (pH)	retention time	compartments simulated (pH)
1	15 min	stomach (pH 1.3)	1 h	stomach (pH 4.9)
2	16 min	duodenum (pH 6)	1 h 12 min	duodenum and jejunum 1 (pH 5.4)
3	1 h 9 min	jejunum 1 and 2 (pH 6.3)	44 min	jejunum 2 (pH 6.0)
4	1h	ileum 1 and 2 (pH 6.7)	1 h	ileum 1 and 2 (pH 6.7)
5	18 min	ileum 3 (pH 7.4)	18 min	ileum 3 (pH 7.4)
6	17 h 26 min	cecum and ascending colon (pH 6.6)	17 h 26 min	cecum and ascending colon (pH 6.6)

Dissolution tests described in Table 3 were conducted in triplicate using the Reciprocating Cylinder Apparatus Bio-Dis Extended-Release Tester (Agilent Technologies Inc., Santa Clara, CA), maintained at 37 ± 0.5 °C. Each vessel line was filled with 250 mL of the described dissolution media. Stainless steel screens (840 μm mesh size) were used in the top caps, polypropylene screens (840 μm mesh size) were used in the bottom caps of the inner sample tubes, and the equipment performed agitation movements adjusted to 5 dpm (dips per minute) for fasted and 30 dpm for fed states. Samples were collected at representative intervals and analyzed as described in Section 2.3.

**Table 3:** USP 3 Methods Employed to Evaluate Pristiq Tablets

method	apparatus	agitation	media
USP 3/5 DPM/fasted gradient	USP 3 (Bio-Dis)	5 dpm	fasted state^a^
USP 3/30 DPM/fed gradient	USP 3 (Bio-Dis)	30 dpm	fed state^a^
USP 3/5 DPM/NaCl 0.9%	USP 3 (Bio-Dis)	5 dpm	NaCl 0.9%
USP 3/30 DPM/NaCl 0.9%	USP 3 (Bio-Dis)	30 dpm	NaCl 0.9%

a Gradient described in Table 2.

### 2.6 Statistical and Mathematical Evaluation of Dissolution Profiles

To perform the statistical analysis, dissolution efficiency (DE%) was calculated for all dissolution profiles obtained using the Microsoft Excel (Microsoft Corporation Inc., Redmond, WA) DDSolver add-in.^22^ The dissolution efficiencies of each replicate were used as the dependent variable to perform the statistical analysis using Statistica software, version 13 (TIBCO Software Inc., Palo Alto, CA). Due to the small sample size (*n* = 3 per group), these analyses were conducted assuming approximate normality. Differences among groups were evaluated using one-way analysis of variance (ANOVA), followed by Tukey’s test. Previously to the ANOVA test, the homogeneity of the variances of the data was met by conducting Levene’s test (*p* > 0.05).

A further analysis was conducted by normalizing the last time point of the method USP 2/50 rpm/NaCl 0.9% with the ones obtained in USP 3, simulating the fasted state. This was made by estimating the amount of drug dissolved in each vessel at the 20.92 h time point. The estimation was based on the ratio of the drug dissolved between 20 and 24h time points. After these data were obtained, ANOVA was conducted as previously described.

To compare the dissolution profiles, the difference (f1) and similarity (f2) factors were also calculated using DDSolver. Additionally, the same add-in was used to evaluate dissolution kinetics of the profiles obtained under USP 3/30 DPM/Fed Gradient and USP 2/50 rpm/NaCl 0.9% methods, by applying two dependent models (Higuchi and Korsmeyer–Peppas). Models were applied to the dissolution profiles, considering only values under 60% drug release, to correctly analyze the mechanism of release.^23,24^ The equations used in the Higuchi and Korsmeyer–Peppas models are displayed below, respectively,^22^

$$F=k_{H}\times t^{0.5}$$

$$F=k_{\mathrm{KP}}\times t^{n}$$

where *F* stands for the fraction (%) of drug released in time *t*; *k*_H_ stands for the Higuchi release constant, *k*_KP_ stands for the Korsmeyer–Peppas release constant, and *n* stands for the diffusional exponent.^22^

## 3 Results and Discussions

### 3.1 In Vitro Dissolution Testing

In vitro dissolution methods were developed and used to obtain Pristiq tablet dissolution profiles and to identify candidates for being biopredictive for the fed state. In this context, for the purpose of enhancing hydrodynamics to create conditions that could better represent the fed state, a biopredictive dissolution method for the fasted state (USP 2/50 rpm/NaCl 0.9%) developed by Carapeto et al.^17^ was adapted by increasing the rotation speed to 75 and 100 rpm. The impact of different dissolution medium pH values was not explored since it showed no influence on the dissolution results in the study that proposed the base method used.^17^

The dissolution profiles of Pristiq tablets are shown in Figure 2 and reveal a very low impact of increasing rotation speed on drug release, confirmed by the difference (f1) and similarity (f2) factor values (Table 4) calculated by comparing these dissolution profiles with the original 50 rpm method (Table 1).

**Figure 2:**
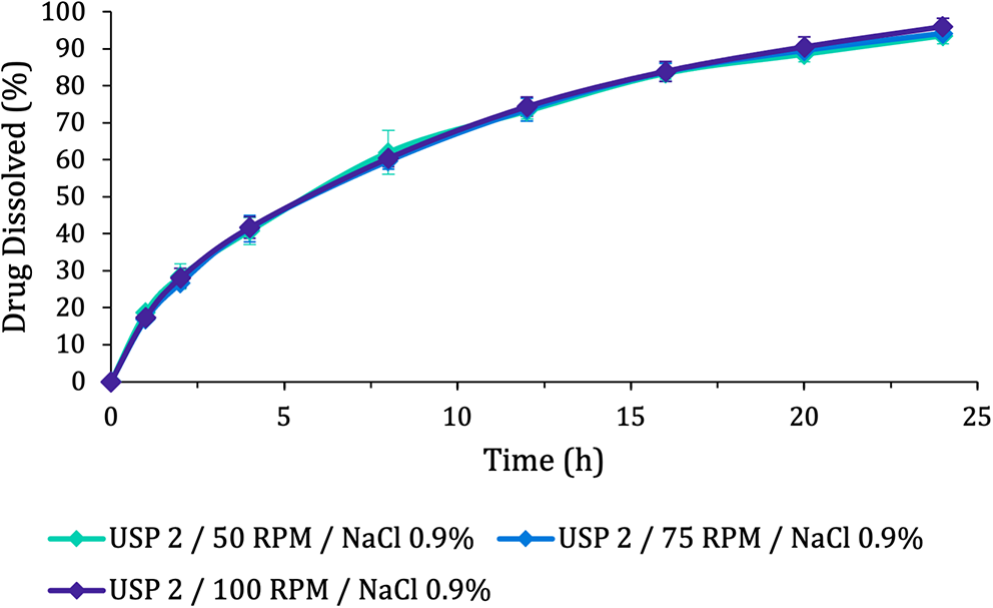
Dissolution profiles of Pristiq tablets obtained under different agitation conditions (50, 75, and 100 rpm). Error bars represent the standard deviation between the replicates.

**Table 4:** Difference and Similarity Factors Calculated by Comparing the Dissolution Profiles Obtained by the 75 and 100 rpm Methods with the Profile of the Original 50 rpm Method

factor	USP 2/75 rpm/NaCl 0.9%	USP 2/100 rpm/NaCl 0.9%
difference (f1)	1.90	2.16
similarity (f2)	89.41	88.42

These results may appear to be controversial at first, since higher rotation speeds would promote higher hydrodynamics and, therefore, should result in a faster dissolution. However, in the context of extended- and sustained-release formulations, this logic may not always be true.

In this regard, Cascone^25^ studied the influence of different USP 2 rotation speed conditions on commercial immediate and extended-release diclofenac formulations, by using the dissolution profile obtained by USP 2 at 50 rpm as the reference data for obtaining f1 and f2 factors, comparing with the other agitation conditions. Their results revealed that the speed increase led to different profiles for only the immediate release formulation.

A factor that may explain this behavior was explored by Ranjan and Jha^26^ in their study on the use of the USP 2 apparatus on the drug release from polymeric controlled-release formulations. The authors observed that the active pharmaceutical ingredient to polymer ratio directly impacted the tendency of the tablet to erode or swell. Furthermore, it is possible to observe that at a lower ratio, where erosion is favored, their tablet showed much more sensitivity to the increase in rotation speed, when compared to a higher ratio, which showed almost no erosion and was visually less impacted by the rotation speed in terms of drug release.

This information is very relevant for the present study, since the desvenlafaxine tablets used are based on the polymeric matrix technology, which contains hypromellose as the extended-release agent,^27^ and, therefore, may also perform as described, especially based on the robust gel formed during dissolution, with very little noticeable erosion.

Since almost no change was observed and given the considerably high agitation already performed for USP 2, the USP 3 apparatus was used as a strategy to promote higher hydrodynamics in the dissolution process, adequately simulating fed conditions. This strategy may be especially promising due to the USP 3 design, which promotes an intense exposure of the entire tablet to the dissolution media, potentially promoting greater erosion, as discussed by Missaghi and Fassihi,^28^ in their study to select an appropriate apparatus for eroding and swelling matrix tablets containing dimenhydrinate. Also, the authors discuss that not having a tablet in a constant position may better resemble the gastrointestinal tract environment, highlighting another advantage for the use of the USP 3 apparatus.

In this context, two different methods were proposed, based on both fasted and fed conditions, varying not only the agitation (5 and 30 dpm) but also the pH gradient and retention time on each vessel row. These conditions were established to specifically correspond to each of the physiological states. Therefore, the agitation speeds were determined based on common parameters used to mimic such conditions, identified as 5–15 dpm for the fasted state and 30–40 dpm for the fed state by Pezzini et al.^10^ In addition, the pH gradient and retention times were defined based on the values used by the GastroPlus software to simulate each of the states (Table 2). This combination resulted in the methods “USP 3/5 DPM/Fasted Gradient” and USP 3/30 DPM/Fed Gradient”.

The dissolution profiles obtained based on these two methods were compared to the original biopredictive fasted method (Figure 3) and revealed a noticeable difference, mainly between the simulated fed method and the other two, which seem quite similar.

**Figure 3:**
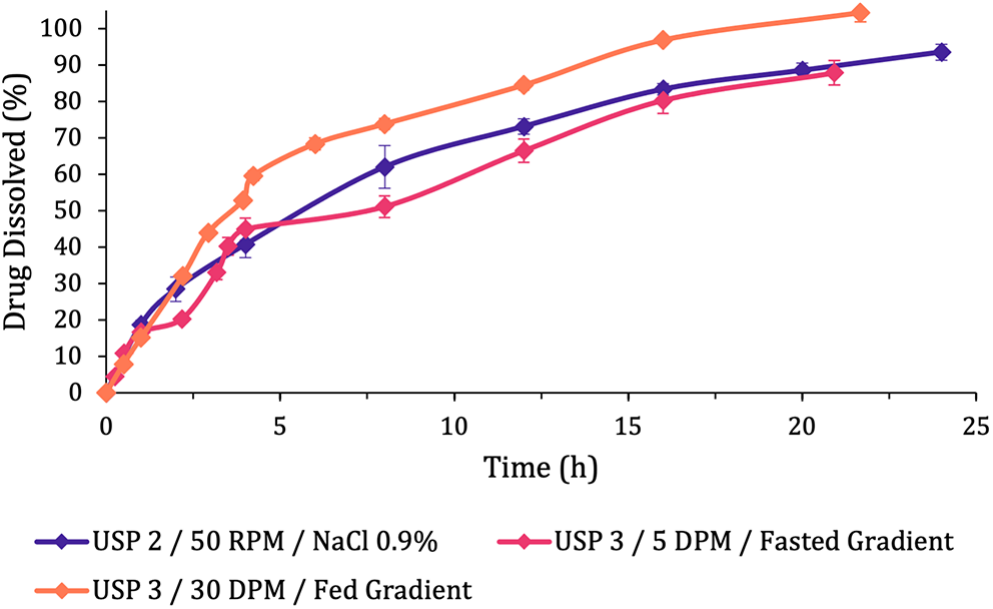
Dissolution profiles of Pristiq tablets obtained under different apparatus and agitation, used to emulate fasted and fed conditions. Error bars represent the standard deviation between the replicates.

Apart from the visual analysis of Figure 3, the statistical ANOVA analysis to compare the dissolution profiles obtained was based on their dissolution efficiencies (DE%), and it was crucial for their differentiation.

In the Tukey test results (Table 5), it is possible to observe differences between the methods, which were categorized into three distinct groups. In this analysis, all dissolution profiles were evaluated, including those based on the USP 2 apparatus that were previously discussed and those USP 3 methods that are discussed in the following subsection.

**Table 5:** Results of the Tukey Test Based on a Confidence Interval of 0.95 Were Obtained after the ANOVA Analysis^a^

method	DE (%) mean	group
USP 3/5 DPM/fasted gradient	58.55	b
USP 3/5 DPM/NaCl 0.9%	59.82	b
USP 2/75 rpm/NaCl 0.9%	66.44	a
USP 2/50 rpm/NaCl 0.9%	66.59	a
USP 2/100 rpm/NaCl 0.9%	67.15	a
USP 3/30 DPM/NaCl 0.9%	73.90	c
USP 3/30 DPM/fed gradient	75.38	c

a Methods within the same group (letters a, b, or c) are considered to have no significant differences in dissolution efficiency.

The Tukey test (Table 5) categorized the fasted biopredictive method under the same category as the USP 2 75 and 100 rpm methods, which is expected based on the high similarity of the results observed in the dissolution profile comparison (Figure 2). The test also grouped the USP 3 fed state method in a different category as a result of the dissolution profile differences observed in Figure 3.

Additionally, the USP 3 fasted state was grouped separately from the USP 2 fasted biopredictive method, which is not expected based on the similarities identified between both dissolution profiles (Figure 3). However, this result may be biased to separate the USP 2 and the USP 3 fasted profiles, since the paddle method is more than 3 h longer than the USP 3 fasted method after a point where the dissolution plateau is close, which reflects on higher DE% values.

Therefore, to standardize the dissolution efficiencies, the 20.92 h time point was estimated for the USP 2 apparatus, and a further ANOVA analysis was made. In this analysis, the Tukey test grouped both methods (Table 6).

**Table 6:** Results of the Tukey Test Based on a Confidence Interval of 0.95, Performed after the ANOVA Analysis of DE%, Considering the Dissolution Efficiency under the Same Final Assay Time (20.92 h) as that for the Dependent Variable^a^

method	DE (%) mean	group
USP 3/5 DPM/fasted gradient	58.55	a
USP 3/5 DPM/NaCl 0.9%	59.82	a
USP 2/50 rpm/NaCl 0.9%	62.91	a

a Methods within the same group (letters a, b, or c) are considered to have no significant differences in dissolution efficiency.

The new results, which classified both methods in the same group, are expected, based on what was observed by Mwila, Khamanga, and Walker,^29^ who studied the effects of agitation on the dissolution rate of Nevirapine sustained-release matrix tablets. In this study, the authors compared the dissolution profiles obtained at several agitation rates, based on the f1 and f2 factors calculated, and found that results on the USP 3 apparatus were closer to USP 2 at 50 rpm when using rates of 5 and 8 dpm.

Also, based on these results, it is possible to infer that the method developed to mimic fasted conditions using apparatus 3 (USP 3/5 DPM/Fasted Gradient) is also biopredictive for this state, since it was shown to be equivalent with the USP 2/50 rpm/NaCl 0.9% method, based on the Tukey test shown in Table 6, which was already confirmed to be biopredictive for the fasted state in a previous study.^17^

Regarding the simulated fed method (USP 3, 30 DPM, and Fed Gradient), a faster dissolution was observed (Figure 3), with higher drug dissolved rates obtained. These results are expected based on the studies conducted by Perivilli, Prevost, and Stippler,^30^ who found that the increases in the dip rate from 5 dpm to 30 dpm led to an increase in velocity magnitude, while retaining most of the flow characteristics identified by the authors. The same phenomenon was described by Wang et al.,^31^ who stated that maintaining the flow field characteristics while increasing liquid velocity and intensity of the disordered flow when increasing dip rate was responsible for promoting drug dissolution.

Furthermore, the fact that increasing the dip rate in the USP 3 apparatus led to a faster dissolution, in opposition to the rotation speed increase in USP 2, may be explained by the dissolution kinetics obtained under each of the apparatuses used. Table 7 summarizes the comparison of USP 3 under fed conditions and USP 2 at 50 rpm profiles.

**Table 7:** Results of the Dissolution Kinetics Obtained for Pristiq under USP 2/50 rpm/NaCl 0.9% and USP 3/30 DPM/Fed Gradient Methods^a^

Higuchi	USP 2/50 rpm/NaCl 0.9%	USP 3/30 DPM/fed gradient
*k*_H_	20.07	3.04
*R*_adj._^2^	0.9881	0.9057
AIC	7.32	33.99
MSC	4.18	1.69

Korsmeyer–Peppas	USP 2/50 rpm/NaCl 0.9%	USP 3/30 DPM/fed gradient
*k*_KP_	19.35	0.33
*n*	0.53	0.94
*R*_adj._^2^	0.9874	0.9933
AIC	5.90	18.45
MSC	4.53	4.28

a Results of the release constants (*k*_H_ and *k*_KP_), adjusted determination coefficients (*R*_adj._^2^), release coefficient (*n*), Akaike criterion (AIC), and model selection criteria (MSC) were obtained.

The results obtained show that the Higuchi model better explains the dissolution kinetics based on the higher *R*_adj._^2^ values when compared to the Korsmeyer–Peppas model for the dissolution profile obtained under the USP 2 apparatus, which reveals a release based mainly on the diffusion process. However, the *R*_adj_^2^ only slightly differs from the one obtained for the Korsmeyer–Peppas, so conclusions based on the release coefficient (*n*) may also be made.^23^

The dissolution profile obtained under the USP 3 method, in turn, was better described by the Korsmeyer–Peppas model, obtaining a high *R*_adj_^2^ value (0.9933). Based on the release coefficient values, the erosion process is favored under the USP 3 at 30 dpm (*n* > 0.89), while a superposition of diffusion and erosion is expected under the USP 2 apparatus (0.45 < *n* < 0.89).^24,32^

In summary, the simulated fed method is, therefore, a candidate for biopredicting the fed state, since the higher agitation promoted, reflected in a faster release, a condition compatible with the biological fed state, which leads to a 16% increase in desvenlafaxine *C*_max_, as described on the Pristiq prescribing information.^33^

### 3.2 Dissolution Media Significance Study

Since desvenlafaxine is a high-solubility drug^14^ and considering that in a previous work, Carapeto et al.^17^ found no difference between profiles obtained under pH-modified dissolution media, as pH 6.8, and a medium composed solely of NaCl 0.9%, it is important to assess if the pH gradients, designed to emulate the fasted and fed states, are being responsible for any of the differences observed and discussed in Section 3.1.

Therefore, to verify the influence of the pH gradients on the Pristiq tablet dissolution, the same methods designed for fasted and fed conditions were replicated, substituting all of the media with a NaCl 0.9% solution but maintaining their original dip rates and retention times.

The dissolution profiles obtained (Figure 4) in this study reveal very close results after substitution of the gradient media. This is also confirmed by the difference and similarity factors obtained by comparing the methods with and without the use of a pH gradient (Table 8).

**Figure 4:**
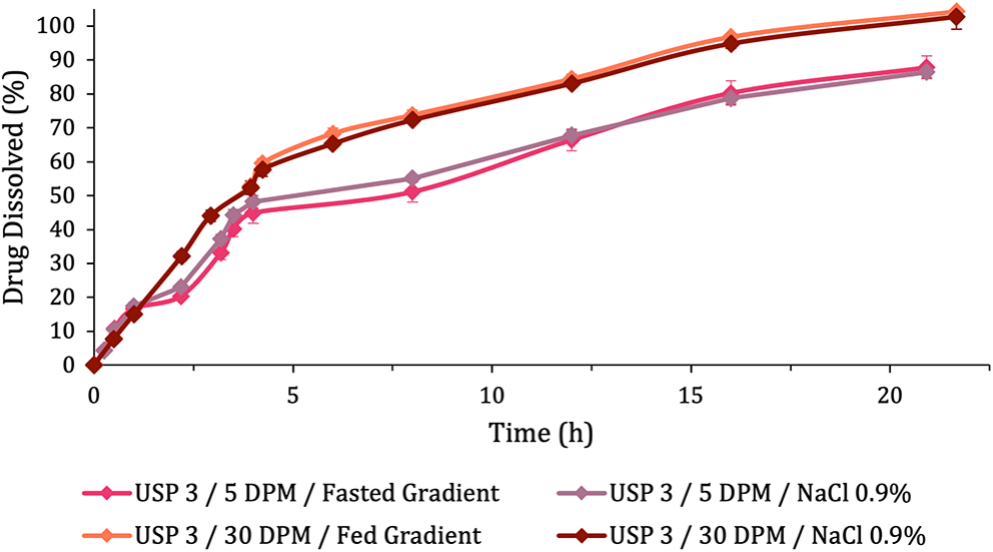
Dissolution profiles of Pristiq tablets obtained under fasted and fed conditions, employing both pH-gradient-based media and exclusively NaCl 0.9% medium. Error bars represent the standard deviation between the replicates.

**Table 8:** Difference and Similarity Factors Calculated by Comparing the Dissolution Profiles Obtained by Fasted and Fed Gradient Methods and Their Alternative Version with NaCl 0.9%-Based Media

factor	USP 3/5 DPM/fasted gradient vs USP 3/5 DPM/NaCl 0.9%	USP 3/30 DPM/fed gradient vs USP 3/30 DPM/NaCl 0.9%
difference (f1)	5.21	1.94
similarity (f2)	78.51	88.20

Apart from the f1 and f2 factors, the statistical analysis of variance also suggests that there is no significant difference in substituting the pH gradients for 0.9% NaCl media, since the profiles with the same agitation speed (5 and 30 dpm) belong to the same Tukey group (Table 5).

These results indicate that desvenlafaxine succinate release from its extended-release formulation is not dependent on the pH of the dissolution media. This is explained by the characteristics of the tablet matrix, which is based on hypromellose, a nonionic polymer, which, as described by Asare-Addo et al.,^34^ can produce a pH-independent release mechanism when the solubility of the drug within the formulation is also pH-independent.

However, as the release is pH-independent, it was not expected that the use of the USP 3 apparatus would be beneficial since its use is mainly focused on the benefits of employing different dissolution media and pH gradients on the same dissolution assay,^35^ which consists of a strategy focused on pH-dependent formulations or drugs.

Therefore, these results show the importance of the use of the USP 3 apparatus since the current study shows that the equipment design may also play an important role in emulating physiological conditions by promoting dissolution hydrodynamics that would allow the establishment of biopredictive methods. These findings are particularly beneficial to aid the development of such methods for high-solubility drugs in robust controlled-release matrices, which is the case for desvenlafaxine tablets.

### 3.3 In Silico Simulations

Besides obtaining the dissolution profiles, to assess the biopredictibility for the fed state of the obtained methods, it is crucial to perform in silico simulations, allowing a comparison of the predicted pharmacokinetic parameters obtained for each of the dissolution profiles with those obtained in in vivo studies.

Therefore, the results obtained through simulating the in vivo behavior of the extended-release tablets containing desvenlafaxine, using each in vitro dissolution profile (Tables 9 and 10), reveal the biopredictive potential of such methods, expressed by the predictive/observed ratio (P/O), which should be between 0.8 and 1.25 to adequately predict a PK parameter.

**Table 9:** Predicted (P) and Observed (O) Results of AUC0-*t* (ng h/mL), Considering the Fed State^a^

method	observed (O)	predicted (P)	P/O
USP 2/50 rpm/NaCl 0.9%	2458.94	2438.00	1.01^b^
USP 2/75 rpm/NaCl 0.9%	2458.94	2305.90	1.07^b^
USP 2/100 rpm/NaCl 0.9%	2458.94	2362.70	1.04^b^
USP 3/5 DPM/fasted gradient	2458.94	2452.60	1.00^b^
USP 3/5 DPM/NaCl 0.9%	2458.94	7078.10	1.18^b^
USP 3/30 DPM/fed gradient	2458.94	2916.50	0.84^b^
USP 3/30 DPM/NaCl 0.9%	2458.94	2931.90	0.84^b^

a Predicted results were obtained from a population simulation (*n* = 43) in the fed state (800 kcal and 50% fat). Observed results reported by Pedrazzoli-Júnior et al.^18^

b Values within the specification to be considered biopredictive for AUC0-*t* (ng h/mL).

**Table 10:** Predicted (P) and Observed (O) Results of *C*_max_ (ng/mL), Considering the Fed State^a^

method	observed (O)	predicted (P)	P/O
USP 2/50 rpm/NaCl 0.9%	127.00	96.54	1.32
USP 2/75 rpm/NaCl 0.9%	127.00	95.09	1.34
USP 2/100 rpm/NaCl 0.9%	127.00	93.11	1.36
USP 3/5 DPM/fasted gradient	127.00	99.86	1.27
USP 3/5 DPM/NaCl 0.9%	127.00	93.70	1.36
USP 3/30 DPM/fed gradient	127.00	127.31	1.00^b^
USP 3/30 DPM/NaCl 0.9%	127.00	126.16	1.01^b^

a Predicted results obtained from a population simulation (*n* = 43) in the fed state (800 kcal and 50% fat). Observed results reported by Pedrazzoli-Júnior et al.^18^

b Values within the specification to be considered biopredictive for *C*_max_ (ng/mL).

One interesting factor is that all methods passed the specification for AUC0-t, as shown in Table 9; therefore, the *C*_max_ value obtained (Table 10) is the determining factor for the dissolution method to be biopredictive for the fed state.

It was observed that only the USP 3 and 30 DPM methods were biopredictive (P/O between 0.8 and 1.25). This not only confirms the application potential of the USP 3 apparatus in developing methods to correctly simulate the fed state but also highlights the independence of the use of pH gradients to simulate this state, in the case of high-solubility drugs, such as desvenlafaxine succinate.

Considering that the increase in rotation speed on USP 2 apparatus did not lead to higher drug release to simulate the fed state and since that it was possible to be achieved by the use of USP 3 with the same dissolution media (NaCl 0.9%), we can assume that the different hydrodynamic promoted by the USP 3 apparatus is an important factor to be considered for desvenlafaxine extended-release tablets, apart from the establishment of a pH gradient.

In the case of desvenlafaxine, it was possible to develop both fasted and fed biopredictive methods exclusively using 0.9% NaCl. Also, the fed state was correctly predicted only by altering the dissolution apparatus from USP 2 to USP 3, proving that it can be an important tool for developing methods that differentiate between physiological fasted and fed states, especially for pH-independent formulations.

## 4 Conclusions

In this work, it was possible to verify the potential of the USP 3 apparatus as a tool for developing fasted and fed biopredictive dissolution methods and demonstrate a new hydrodynamic-driven approach for its use, since it may be especially important for high-solubility drugs in robust extended-release matrices. Regarding specifically the desvenlafaxine extended-release tablets, it was possible to develop fasted and fed state biopredictive methods conducted in the USP 3 apparatus. Additionally, the attainment of conditions compatible with agitation in the fed state was also possible only on the USP 3 apparatus, revealing huge advantages of its use in this context. Finally, an independence of pH gradients was identified for the tablets, mainly because of the combination of the high solubility of the drug in physiological conditions with its incorporation in a nonionic matrix, as is the case with hypromellose.

## Abbreviations

PBBM: physiologically based biopharmaceutics modeling
dpm: dips per minute
rpm: rotations per minute
DE%: dissolution efficiency
